# E3 Ligase Activity of XIAP RING Domain Is Required for XIAP-Mediated Cancer Cell Migration, but Not for Its RhoGDI Binding Activity

**DOI:** 10.1371/journal.pone.0035682

**Published:** 2012-04-19

**Authors:** Jinyi Liu, Dongyun Zhang, Wenjing Luo, Jianxiu Yu, Jingxia Li, Yonghui Yu, Xinhai Zhang, Jingyuan Chen, Xue-Ru Wu, Chuanshu Huang

**Affiliations:** 1 Nelson Institute of Environmental Medicine, New York University School of Medicine, Tuxedo, New York, United States of America; 2 Department of Occupational and Environmental Health Sciences, Fourth Military Medical University, Xi'an, Shanxi, China; 3 Departments of Urology and Pathology, New York University School of Medicine, Tuxedo, New York, United States of America; Rush University Medical Center, United States of America

## Abstract

Although an increased expression level of XIAP is associated with cancer cell metastasis, the underlying molecular mechanisms remain largely unexplored. To verify the specific structural basis of XIAP for regulation of cancer cell migration, we introduced different XIAP domains into XIAP^−/−^ HCT116 cells, and found that reconstitutive expression of full length HA-XIAP and HA-XIAP ΔBIR, both of which have intact RING domain, restored β-Actin expression, actin polymerization and cancer cell motility. Whereas introduction of HA-XIAP ΔRING or H467A mutant, which abolished its E3 ligase function, did not show obvious restoration, demonstrating that E3 ligase activity of XIAP RING domain played a crucial role of XIAP in regulation of cancer cell motility. Moreover, RING domain rather than BIR domain was required for interaction with RhoGDI independent on its E3 ligase activity. To sum up, our present studies found that role of XIAP in regulating cellular motility was uncoupled from its caspase-inhibitory properties, but related to physical interaction between RhoGDI and its RING domain. Although E3 ligase activity of RING domain contributed to cell migration, it was not involved in RhoGDI binding nor its ubiquitinational modification.

## Introduction

The X-linked inhibitor of apoptosis protein (XIAP) is a member of the inhibitors of the apoptosis protein (IAP) family [1]. XIAP was first recognized by its potent properties in regulating cell apoptosis [2], [3]. Later investigations found that XIAP may regulate other cellular pathways uncoupled from its caspase-inhibitory activities [4], [5], majorly inspired by the findings from XIAP-deficient mice which displayed no overt apoptotic phenotype [6]. Recently a wide variety of evidence has suggested that the involvements of XIAP in copper metabolism [7], cell motility [8], [9] and activation of JNK and NFκB pathways [10], [11] were unrelated to its inhibitory effect on caspases.

The multiple functions of XIAP root from its structural basis. XIAP is composed of three baculoviral IAP repeat (BIR) domains at amino-terminus and one carboxyl-terminal RING domain [12]. Each BIR domain consists of approximately 70 amino acids that coordinate a zinc ion via histidine and cystein residues [13]. Its potent anti-apoptotic properties are mainly dependent on the functions of a groove in the BIR3 domain and two surfaces on the BIR2 domain which have been reported to bind and inhibit caspase-9 and caspase-3/7 respectively [14]. RING domain is defined by the presence of seven cysteins and one histidine that form cross brace architecture and coordinate two zinc ions [15]. RING domains often function as modulates that confer ubiquitin ligase (E3) activity [13]. By mutating the key histidine residue at amino acid 467 to alanine of human XIAP, Lewis et al found that E3 ubiquitin ligase function of RING was required for the activation of NFκB, while not for Smad-dependent transcription [16], indicating that structure-based functions of XIAP are also cellular context dependent.

Increased expression of XIAP is found in many cancer tissues and associated with chemoresistance, disease progression and poor prognosis [9], [17], [18], [19], [20], [21], [22]. The recent findings from our laboratory and others' demonstrated that XIAP could regulate tumor metastasis [8], [23], [24]. Tumor metastasis is a major cause of death for most cancer patients [25]. Many molecules involved in metastatic cascade are controlled by the members of Ras-superfamily of small GTP-binding proteins, which are able to bind GDP/GTP and hydrolyze GTP leading to activation of downstream effector proteins [26]. Human Rho-GTPase subfamily comprises 23 signaling molecules, among which RhoA, RhoB, Rac1 and Cdc42 are most extensively investigated and reported to control various aspects of cellular motility and invasion, i.e., cellular polarity, ctyoskeletal organization, and signal transduction [27], [28].

Rho-GTPase activity is tightly controlled by four key components involved in GDP/GTP-bound GTPase cycle, including GTPase-activating proteins (GAPs), GDP-dissociation inhibitors (GDIs), GDI dissociation factors (GDFs), and guanine nucleotide exchange factors (GEFs) [29]. RhoGDI plays a key role in balancing the entire GTPase cycle by preventing GDP dissocation and maintaining GTP association through interaction with the prenylation group of GTPase. Thus, it sequesters GTPase in the cytoplasm while localization to the inner plasma membrane is necessary for GTPase activation. The inhibitory effects of RhoGDI on GTPase activity have been supported by several lines of evidence [30], [31], [32]. For instance, Leffers *et al*. have found that overexpression of RhoGDI in human keratinocytes caused disruption of actin cytoskeleton and inhibition of motility [32]. Therefore, RhoGDI is regarded as an attractive candidate for regulating the activity of Rho GTPase in cancer treatment [26].

Our recent studies have proven that XIAP mediated cancer cell motilities via RhoGDI-dependent manner in regulation of cytoskeleton [23]. In the current study, we further elucidated the molecular mechanisms underlying XIAP-RhoGDI protein interaction and provided the structural basis of XIAP for the contribution to mediation of cancer cell motility.

## Results

### RING domain was required for XIAP-mediated β-Actin expression

**Figure 1:**
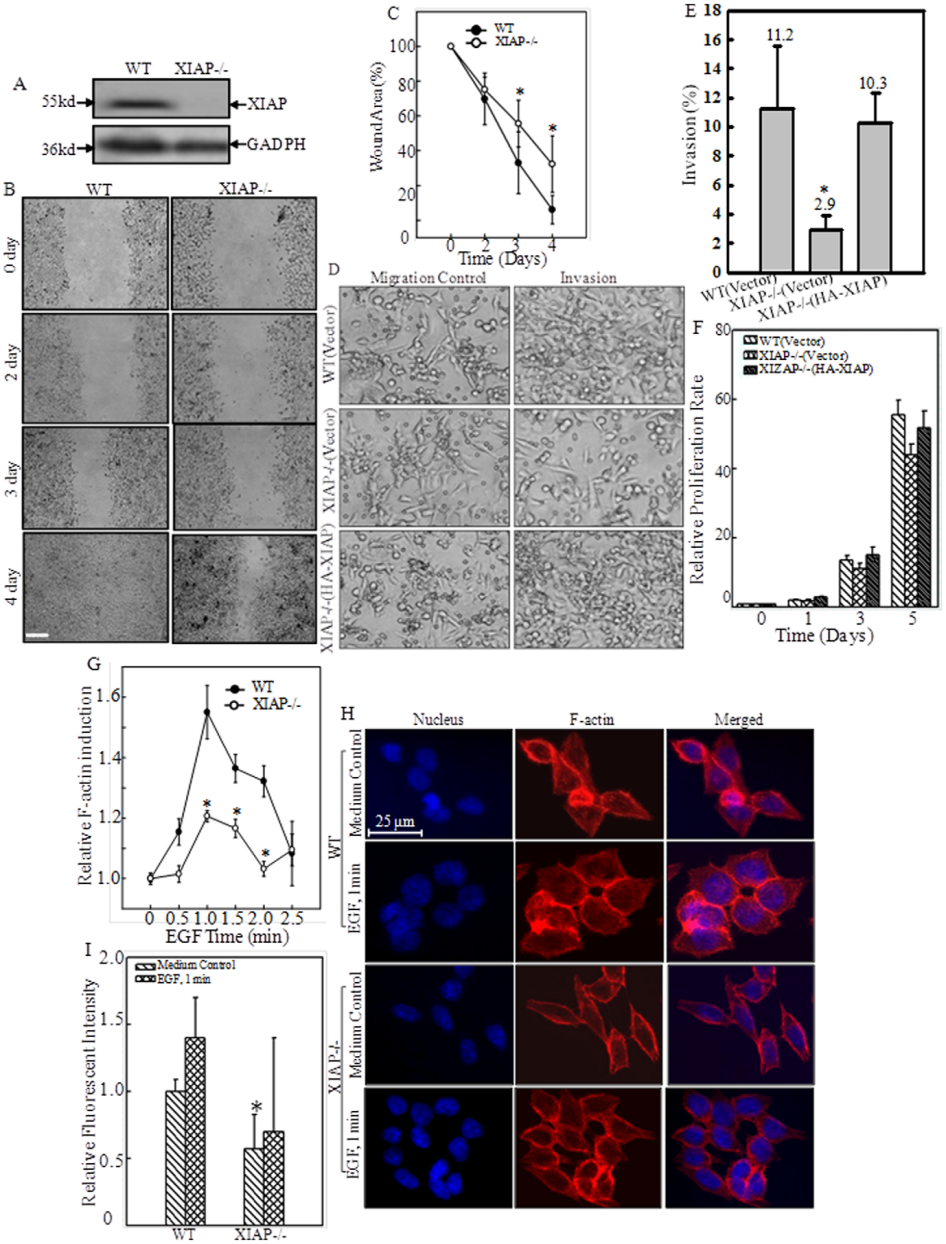
XIAP promoted HCT116 cell migration and invasion. (A), Knockout of XIAP in HCT116 cells was verified by Western Blotting assay. (B and C), Cell migration behavior was evaluated during performance of a wound-healing assay, and images were taken at different time points. Scale bar was 300 µm. The wound area was quantified using Cell Migration Analysis software, and the quantitative data were shown as indicated (error bar represent S.D, n = 3). The asterisk (*) indicates a significant difference in wound area percentage between the indicated cell lines (*p*<0.05). (D and E), Invasion of WT(Vector), XIAP^−/−^(Vector), and XIAP^−/−^(HA-XIAP) HCT116 cells was determined, quantified and expressed as percentage of invasion. Results were represented by the mean ± S.D. of the data from three-independent experiments with duplicate wells for each experiment. The asterisk (*) indicates a significant decrease in invasion percentage compared with that in WT(vector) and XIAP^−/−^(HA-XIAP) cells (*p*<0.01). (F), The proliferate rates of the indicated cell lines were assessed by a CellTiter-Glo® Luminescent Cell Viability Assay kit. Results were represented by the mean ± S.D. of the triplicate wells. (G–I), The indicated cells were treated with or without EGF and F-actin induction was analyzed by spectrophotometer (G), or observed under confocal microscope (H), respectively. The fluorescence of cells was quantified by the software of ImageJ (I). The quantitative data was shown as indicated (error bar represent S.D, n = 3). The asterisk (*) indicates a significant decrease compared with that in WT cells (*p*<0.01).

**Figure 2:**
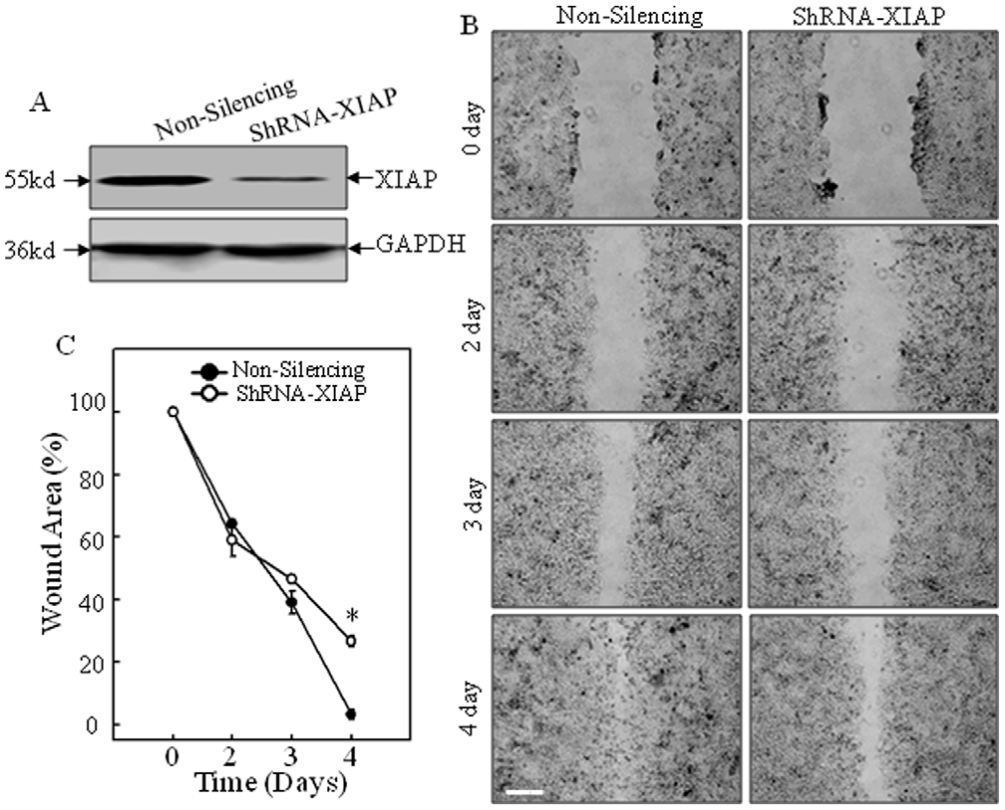
The requirement of XIAP for cell motility was confirmed by knocking down approach. (A), Knockdown of XIAP in HCT116 cells were verified by Western Blotting assay. (B and C), Cell migration behavior was evaluated during performance of a wound-healing assay, and images were taken at different time points. Scale bar was 300 µm. The wound area was quantified using Cell Migration Analysis software, and the quantitative data were shown as indicated (error bar represent S.D, n = 3). The asterisk (*) indicates a significant difference in wound area percentage between the indicated cell lines (*p*<0.05).

XIAP expression is elevated in many cancer cell lines and closely related to the progression and aggression of malignant cancer [33], [34]. Our recent work demonstrated that XIAP could regulate β-Actin expression [23]. As a result, depletion of XIAP expression attenuated cell migration rate and invasive capability as shown in wound healing assay and trans-well assay, respectively (Figs. 1A–1E). To note, there was only marginal difference in proliferation rate between WT and XIAP^−/−^ cells when cultured in normal cell culture medium (10% FBS) for up to 5 days, which included the time range for wound healing assay (Fig. 1F), indicating that the reduced cell migration rate observed in XIAP^−/−^ cells was not due to defective cell proliferation. Moreover, the dynamic induction of actin polymerization, namely F-Actin formation, by EGF was also dramatically reduced in XIAP^−/−^ cells detected by spectrophotometer (Fig. 1G). Consistently, a clear change of cell skeleton morphology and more peripheral ruffles/membrane ruffles were observed in EGF-treated WT HCT116 cells but not in XIAP^−/−^ cells (Figs. 1H & 1I). These phenomena were reproducible by knocking down XIAP in HCT116 cells (Fig. 2). Therefore it indicated that XIAP played a key role in mediation of cancer cell migration and invasion.

**Figure 3:**
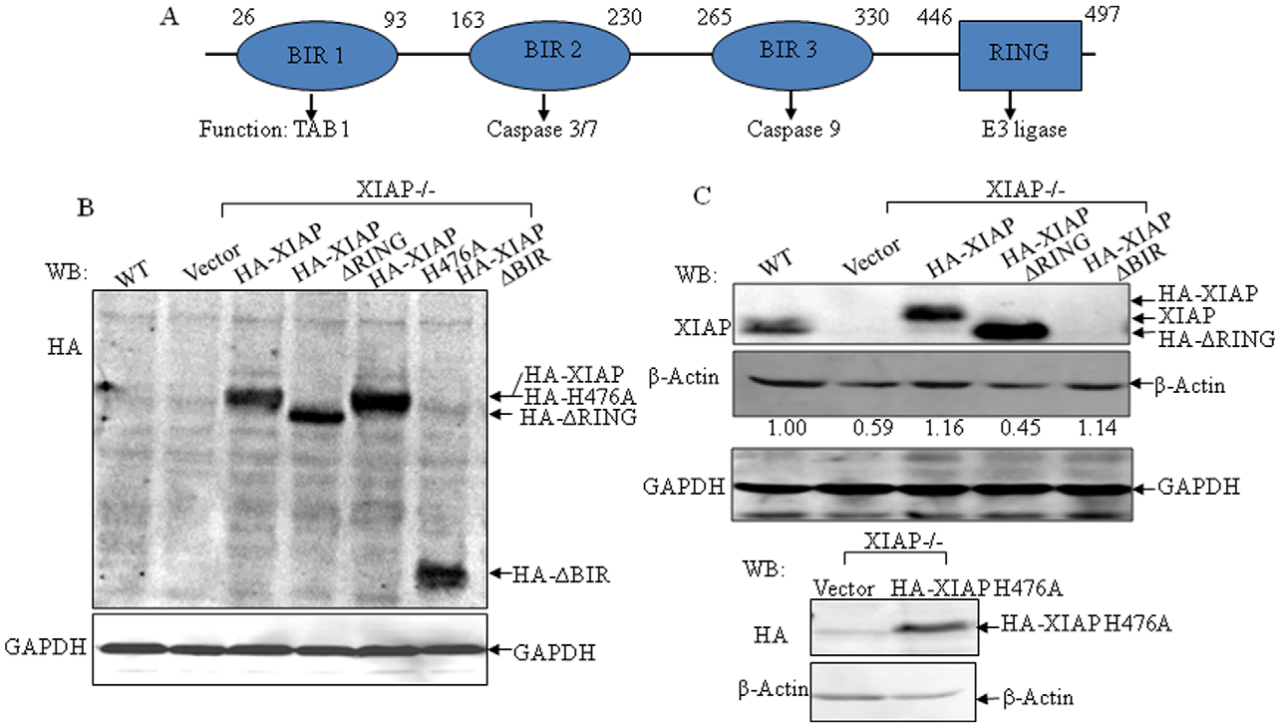
Various XIAP domains were reconstitutivly expressed into XIAP^−/−^ cells. (A), Schematic representation of XIAP protein and identified function of each domain. (B and C), Identification of the stable transfectants harboring XIAP and its various deletion plasmids in XIAP^−/−^ HCT116 cells. The numbers under the bands indicated the densitometric analysis of relative ratios of β-Actin levels to loading controls (GAPDH) evaluated by software of ImageQuant Version 5.2 (Molecular Dynamics, Sunnyvale, CA). Results were representative of at least three independent experiments.

XIAP protein contains four functional domains, including three BIRs and a RING domain (Fig. 3A). The anti-apoptotic function of XIAP BIRs was reported to be attributable to their binding and impairment of caspase activation [1]. The RING domain of XIAP belongs to E3 ligase and mediates protein ubiquitination and degradation [1]. To verify the specific structural basis of XIAP for regulation of cancer cell migration, we transfected different HA-tagged XIAP cDNA constructs, including full-length (HA-XIAP), RING domain-deletion (HA-XIAPΔRING), total BIR deletion (HA-XIAPΔBIR), and a point mutation H467A, which results in loss of E3 ubiquitin ligase activity, into XIAP^−/−^ cells respectively, and the stable transfectants were identified (Fig. 3B). Re-constitutional expression of HA-XIAP or HA-XIAP ΔBIR that contains RING domain into XIAP^−/−^ cells resulted in an increase in β-Actin expression as compared to that in XIAP^−/−^ (Vector) cells, while expression of HA-XIAP ΔRING that contains BIR domains, or HA-XIAP H467A that renders abolishment in E3 ligase activity, did not provide comparable restoration (Fig. 3C). Therefore, it demonstrated that XIAP RING domain and its E3 ligase activity played a role in regulation of β-Actin expression.

### E3 ligase activity of XIAP RING domain was involved in mediation of cell migration and actin polymerization

**Figure 4:**
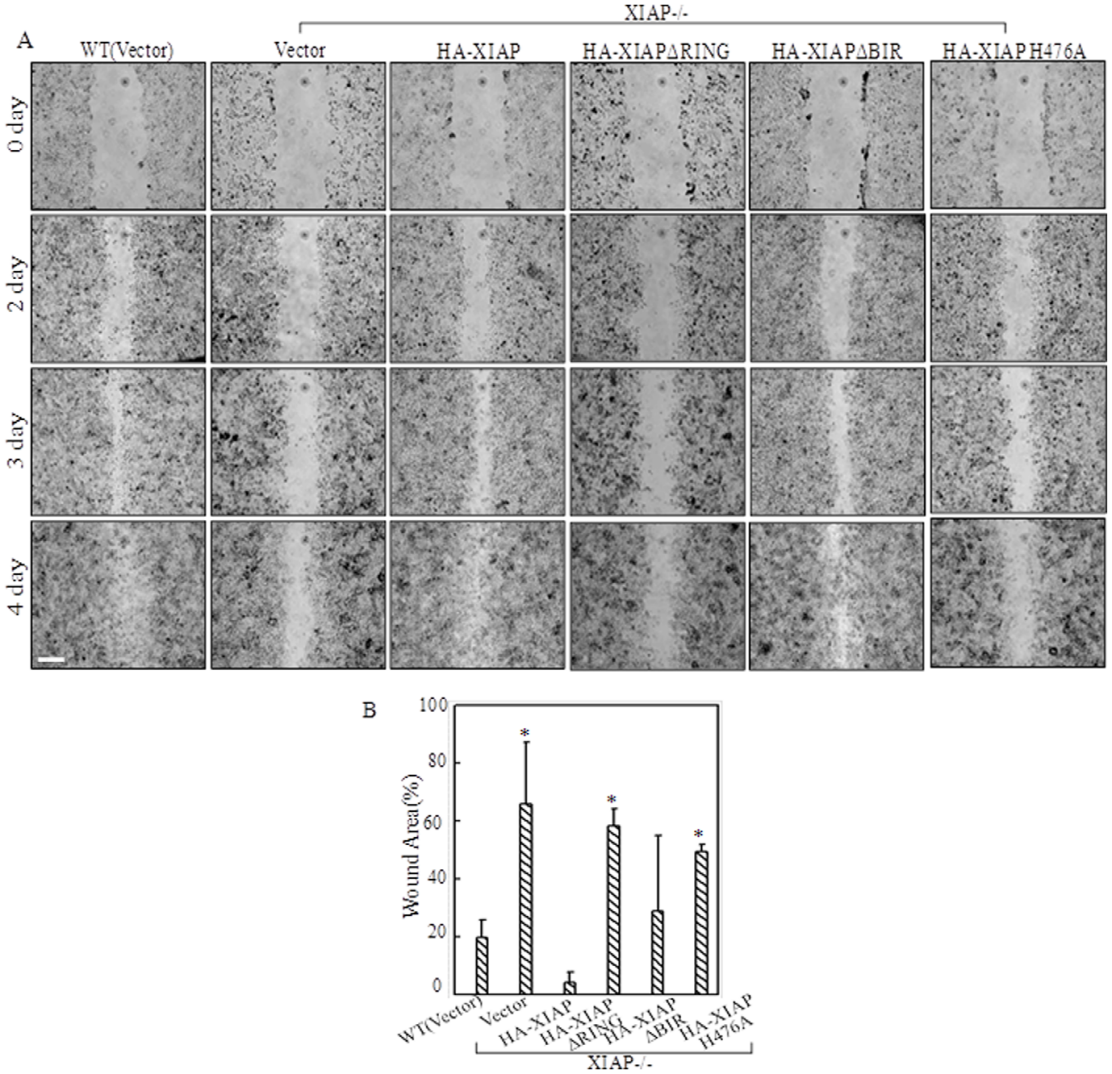
Different XIAP domains involved in cell migration disparately. (A), Cell migration behavior was evaluated with a wound-healing assay, and images were taken at different time points. Scale bar was 300 µm. (B), The wound area left un-closed on the 4^th^ day was quantified using Cell Migration Analysis software, and the quantitative data was shown as indicated (error bar represent S.D, n = 2). The asterisk (*) indicates a significant difference in percentage of wound area compared with that in WT (Vector) cells (*p*<0.05).

To further explore the biological relevance of β-Actin expression change regulated by XIAP RING domain, wound healing assay was performed to compare the migration rates among various transfectants carrying different domains of XIAP as identified in Fig. 3B. In accordance with defects in β-Actin expression, introduction of neither HA-XIAP ΔRING nor HA-XIAP H467A could reverse the impairment in cell migration of XIAP^−/−^ cells, while expression of full length HA-XIAP or HA-XIAPΔBIR, both of which hold intact RING domain, restored the reduction of cell migration capability caused by XIAP depletion (Fig. 4A). While due to the relative low expression of HA-XIAPΔBIR in comparison to that of HA-XIAP in the individual transfectants (Fig. 3B), the wound healing rate observed in HA-XIAPΔBIR-expressing transfectants was slower than that in HA-XIAP-expressing cells (Fig. 4A). The percentage of wound area left un-closed on 4^th^ day compared with that on 0 day was quantified using Cell Migration Analysis software, which showed that the wound areas in XIAP^−/−^(vector), HA-XIAP ΔRING and HA-XIAP H467A transfectants were markedly higher than that in WT HCT116 cells (Fig. 4B). Therefore, it suggested that E3 ligase activity of RING domain played an important role in XIAP-mediated cell motility.

**Figure 5:**
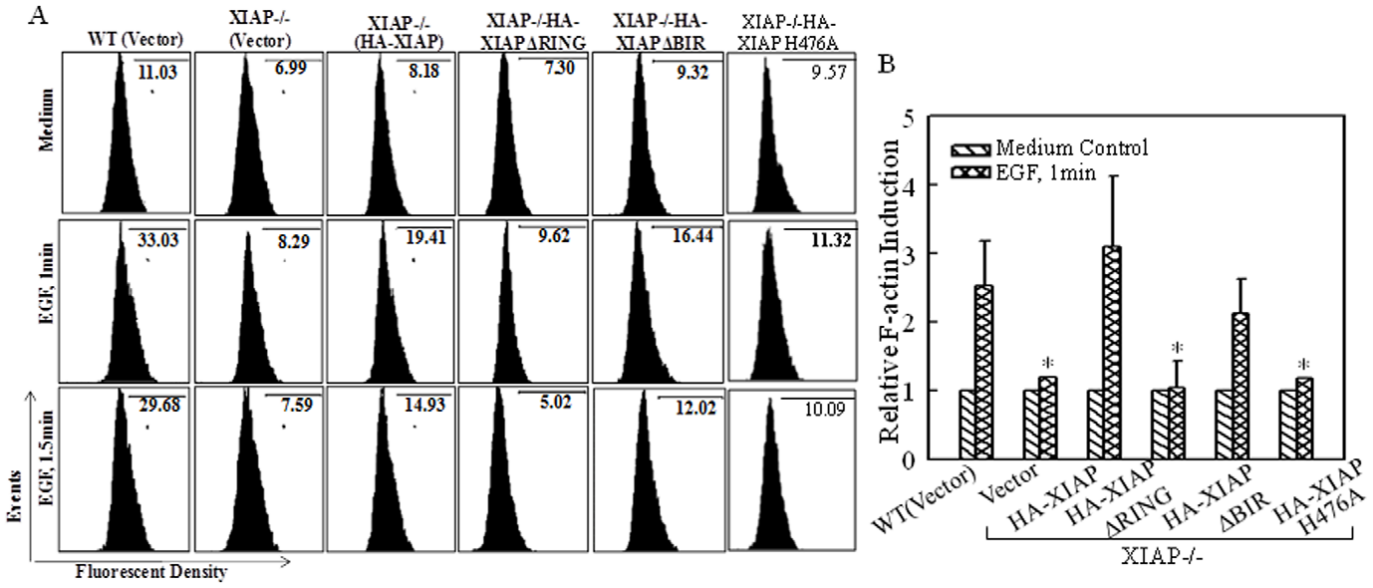
F-actin induction by EGF was regulated differently by various XIAP domains. (A), The indicated cells were treated with or without EGF and F-Actin induction was analyzed by flow cytometry. (B), The quantitative data was shown as indicated (error bar represent S.D, n = 2). The asterisk (*) indicates a significant difference in F-actin induction compared with that in WT(Vector) cells (*p*<0.05).

Actin filaments play a central role in numerous cellular functions, such as cell migration and morphological regulation [35]. To determine potential involvement of different domains of XIAP in regulation of actin polymerization, we treated cells with EGF, and then extracted cells for determination of F-actin levels by flow cytometry using the stable transfectants mentioned above. Again, F-actin formations induced by EGF treatment were obviously obtained in WT HCT116 cells, XIAP^−/−^(HA-XIAP) and XIAP^−/−^(HA-XIAP ΔBIR) transfectants, whereas there was no observable F-actin induction in XIAP^−/−^(vector), XIAP^−/−^(HA-XIAP ΔRING) or XIAP^−/−^(HA-XIAP H467A) transfectants (Fig. 5A). The quantification result was shown in Fig. 5B. Taken together, our results demonstrated that function of XIAP RING domain in regulation of actin polymerization and cell motility was mediated by its E3 ligase activity.

### XIAP RING Domain interacted with RhoGDI, independent on its E3 ligase activity

**Figure 6:**
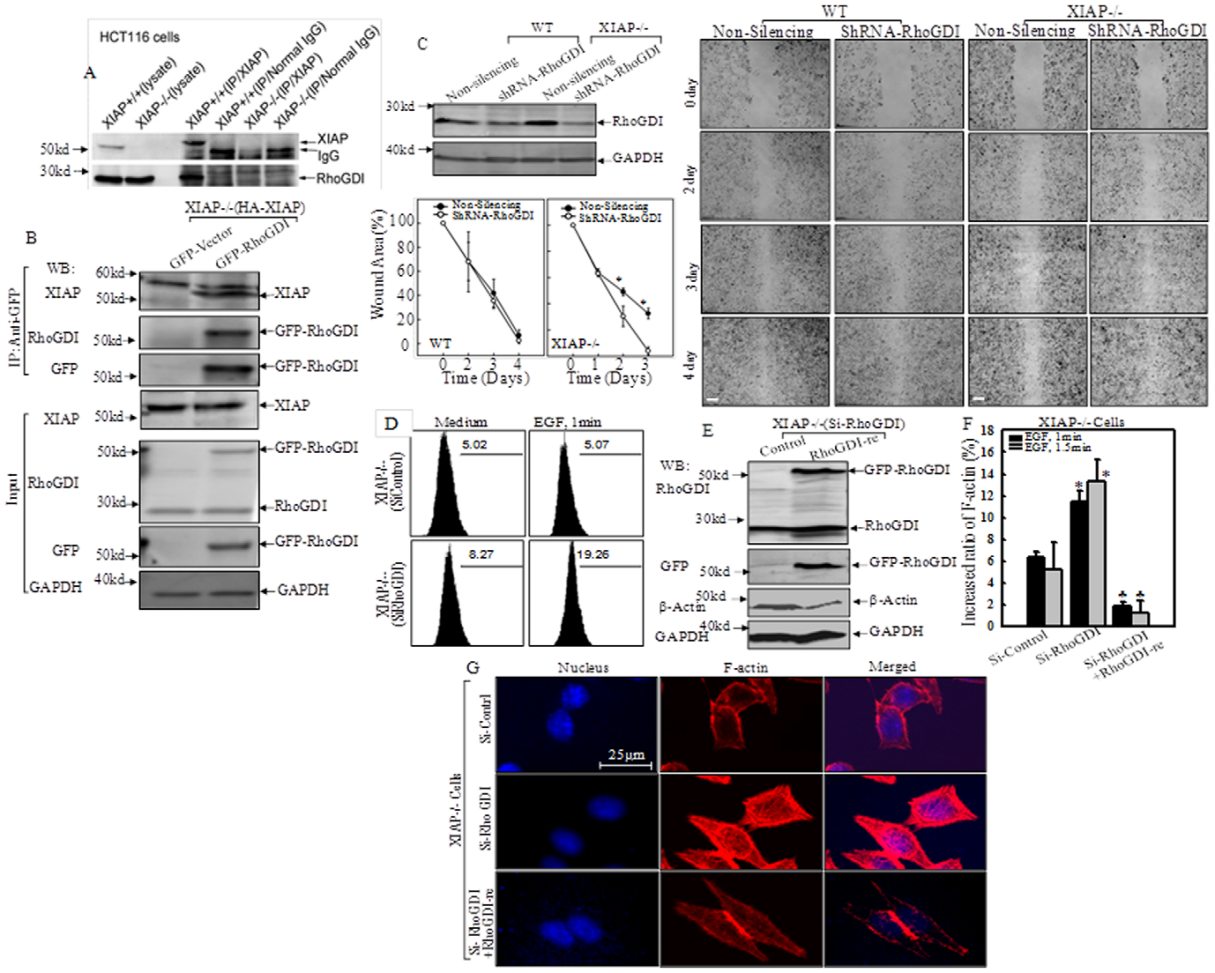
RhoGDI was involved in XIAP regulation of cell migration and actin polymerization. (A), Lysates from WT and XIAP^−/−^ HCT116 cells were Co-immunoprecipitated with anti-XIAP (mouse) antibody or normal mouse IgG, and immunoprecipitates were then subjected to immunoblotting with anti-RhoGDI (rabbit) or anti-XIAP (rabbit) antibodies. Five percent of lysates was used as input. (B), XIAP^−/−^(HA-XIAP) cells were transiently transfected with the GFP-RhoGDI or empty vector, GFTP-Vector. Co-immunoprecipitation was performed with anti-GFP antibody-conjugated agarose beads. Immunoprecipitates were then subjected to immunoblotting using antibodies as indicated. (C). Stable transfectants of shRNA-RhoGDI in WT and XIAP^−/−^ cells were identified. Cell migration was determined by wound healing assays at the indicated times between Non-silencing and shRNA-RhoGDI transfectants in WT and XIAP^−/−^ cells respectively. The wound area was quantified using Cell Migration Analysis software, and the quantitative data was shown as indicated (error bar represent S.D, n = 3). The asterisk (*) indicates a significant difference between the indicated cell lines (*p*<0.05). Scale bar was 300 µm. (D), The indicated cells were treated with EGF for 1 min for determination of F-Actin induction by flow cytometry. (E), Constitutive expression of GFP-RhoGDI-Re in XIAP^−/−^(Si-RhoGDI) was verified by Western Blotting. (F and G), Relative induction of F-Actin in the presence of EGF was determined by spectrophotometer (F), and levels of filamentous Actin were observed under confocal microscopy (G) in the indicated transfectants. The asterisk (*) indicates a significant increase in comparison to those in XIAP^−/−^(Si-Control) (*p*<0.05), and the (♣) indicates a significant decrease in comparison to those in XIAP^−/−^(Si-RhoGDI) cells (*p*<0.001, n = 3).

Our recent work demonstrated that RhoGDI was involved in actin polymerization regulated by XIAP [23]. Therefore we detected the physical interaction between these two molecules by co-immunoprecipitation utilizing anti-XIAP specific antibody. The results showed that RhoGDI was detected in the co-immunoprecipitated complex in XIAP^+/+^, but not XIAP^−/−^ HCT116 cells (Fig. 6A), suggesting that RhoGDI might interact with endogenous XIAP. The interaction between XIAP and RhoGDI was further verified reciprocally by detection of XIAP in co-immunoprecipitation complex pulled-down by anti-GFP antibody using transfectants of XIAP^−/−^(HA-XIAP/GFP-RhoGDI), whereas there was no detectable level of XIAP in Co-IP complex in transfectants of XIAP^−/−^ (HA-XIAP/GFP-vector) (Fig. 6B). Then we knocked down RhoGDI in WT and XIAP^−/−^ cells to confirm the participation of RhoGDI in cell motility. Wound healing assay results showed that knockdown of RhoGDI in WT cells did not cause an obvious change in wound closure rate, however a remarkably increased cell migration was observed in XIAP^−/−^(shRNA-RhoGDI) cells in comparison to that in non-silencing control, XIAP^−/−^(Non-silencing) cells (Fig. 6C). Consistently, knockdown of RhoGDI expression also increased F-actin content in XIAP^−/−^(Si-RhoGDI) cells exposed to EGF (Fig. 6D, p<0.05). The sequences of the RhoGDI gene (401–419), that was complementary to siRNA oligonucleotide in pEGFP-C3/RhoGDI-re construct, were mutated to prevent destruction of exogenous mRNA by RhoGDI siRNA [36]. As shown in Fig. 6E, overexpression of pEGFP-C3/RhoGDI-re was identified in XIAP^−/−^(Si-RhoGDI+RhoGDI-re). This reconstitutive expression of RhoGDI in XIAP^−/−^(Si-RhoGDI+RhoGDI-re) dramatically attenuated actin polymerization induced by EGF treatment in comparison to that in XIAP^−/−^(Si-RhoGDI) cells (2% vs. 14%, p<0.01, Fig. 6F). Moreover, reconstitutive expression of RhoGDI in XIAP^−/−^(Si-RhoGDI+RhoGDI-re) cells restored inhibitory role of RhoGDI on filamentous actin formation (Fig. 6G), suggesting that reintroduction of RhoGDI-re enabled compensation for loss of endogenous RhoGDI function on actin polymerization in XIAP^−/−^ cells. Our results provided evidence that RhoGDI might be involved in XIAP RING domain-mediated regulation of actin polymerization and cell migration.

**Figure 7:**
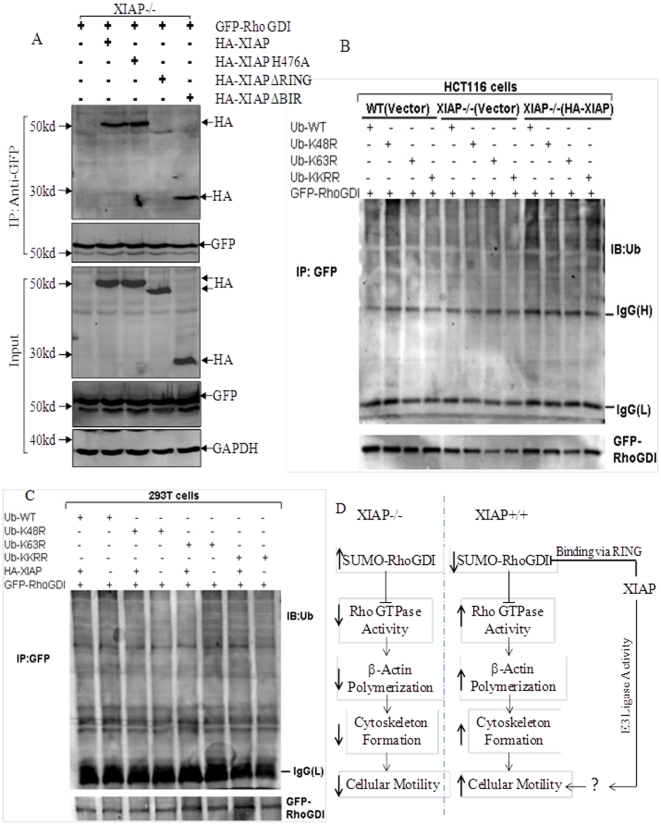
XIAP RING Domain was Responsible for RhoGDI Interaction Independent on its E3 Ligase Activity. (A), XIAP^−/−^ cells were transfected with GFP-RhoGDI, along with HA-XIAP, HA-XIAP H467A, HA-XIAP ΔRING, or HA-XIAP ΔBIR. Co-immunoprecipitation was performed with anti-GFP antibody-conjugated agarose beads. Immunoprecipitates were then subjected to immunoblotting for detection of XIAP using HA antibody. (B). WT(Vector), XIAP^−/−^(Vector) and XIAP^−/−^(HA-XIAP) HCT116 cells were transfected with constructs of GFP-RhoGDI in combination with Ubiquitin-WT, Ubiquitin-K48R, Ubiquitin-K63R or Ubiquitin-K48R/K63R (KKRR). Forty-eight hours after transfection, cells were lysed and co-immunoprecipitated with anti-GFP antibody, and then immunoblotted with anti-Ub and anti-GFP antibodies. (C), 293T cells were transfected with various constructs as indicated for detection of RhoGDI ubiquitination by anti-Ub and anti-GFP antibodies. (D), A model for XIAP-regulated modulation of cell motility: XIAP binds to RhoGDI through its RING domain and inhibits RhoGDI SUMOylation which results in down-regulation of RhoGDI's function and promotes actin polymerization and cell motility. Or E3 ligase activity of XIAP RING domain could regulate some un-verified factors which subsequently control cell migration independent of RhoGDI binding.

To determine specific XIAP domains involved in interaction with RhoGDI protein, we co-transfected GFP-RhoGDI construct with HA-XIAP, HA-XIAP H467A, HA-XIAP ΔRING and HA-XIAP ΔBIR respectively, into XIAP^−/−^ cells. As shown in Fig. 7A, HA-tag was detected in the co-immunocomplex pulled down by anti-GFP antibody in transfectants harboring HA-XIAP and HA-XIAP ΔBIR. Moreover, similar affinity to GFP-RhoGDI was observed in transfectants of HA-XIAP H467A, a mutation with loss of E3 ligase activity in RING domain. While only a marginal band of HA was observed in the immunocomplex from HA-XIAP ΔRING transfectants, revealing that XIAP RING domain played a role in interaction with RhoGDI independent on its E3 ligase activity. In addition, although RING domain of XIAP could bind to RhoGDI, their interaction did not result in ubiquitination of RhoGDI (Figs. 7B and 7C). Conjugation of ubiquitin to RhoGDI was barely detected even in the presence of exogenous wild type ubiquitin in the immunocomplex pulled down by GFP which was tagged to RhoGDI (Fig. 7B). Neither did expressing mutant of ubiquitin render any obvious reductions in ubiquitination of RhoGDI (Fig. 7B). Also there was no observable difference in RhoGDI ubiquitination among WT cells and XIAP^−/−^ cells (Fig. 7B). The similar findings were reproduced in 293T cells as shown in Fig. 7C. Therefore, it was suggested that although E3 ligase activity was required for XIAP-mediated cell migration, it was not essential for RhoGDI binding, neither for its ubiquitinational modification.

## Discussion

Our previous findings have demonstrated that either knockout or knockdown of XIAP decreased HCT116 cell migration and invasion [23]. In the present study, we provided the structural basis of XIAP for its regulatory functions in cancer metastasis. By introducing different XIAP domains into XIAP^−/−^ cells, our work showed that RING domain rather than BIR domain was required for β-Actin expression, cell migration as well as RhoGDI interaction. E3 ligase activity of RING domain contributed to the first two effects, but was not involved in RhoGDI binding nor its ubiquitinational modification, indicating that role of XIAP in regulating cellular motility was uncoupled from its caspase-inhibitory properties, but related to its RING function which was partly attributable to physical interaction with RhoGDI (Fig. 7D).

In wound healing assay, we found that reconstitutive expression of full length HA-XIAP and HA-XIAP ΔBIR, both of which have RING domain, into XIAP^−/−^ HCT116 cells restored cancer cell motility, whereas introduction of HA-XIAP ΔRING or H467A mutant, which abolished its E3 ligase function, did not show obvious restoration, demonstrating that E3 ligase activity of XIAP RING domain played a role in XIAP regulation of cancer cell motility. Alterations in β-Actin levels aroused by expressing various domains of XIAP were consistent with their effects in cell migration. The chief intracellular “motor” of cell migration is actin cytoskeleton [37]. Previous studies suggested that EGF induced cell migration by reorganization of actin cytoskeleton and massive accumulation of F-actin [38]. In our studies, malfunction of actin polymerization in XIAP^−/−^ cells could be rescued by re-constitutional expression of either full length HA-XIAP or HA-XIAP ΔBIR, while overexpression of HA-XIAP ΔRING or H467A showed none of those restorations. In agreement of our findings, Mehrotra's unpublished data also acclaimed that E3 ligase activity of XIAP was critical for its regulatory role in cell metastasis based on the observation that H467A XIAP mutant failed to synergize with survivin in stimulating NFκB-dependent pathway [24]. Therefore, it was clear that function of XIAP in regulation of cell migration was dependent on its E3 ligase activity of RING domain rather than related with its anti-apoptotic potentials.

It has been suggested that role of IAPs in cell motility may be evolutionary conserved since the *Drosophila* IAP homolog DIAP1 has been implicated in cell migration and morphogenesis by controlling non-apoptotic caspase activity [13]. DIAP1 has been shown to promote follicle cells migration within the egg chamber during *Drosophila* oogenesis via regulating activity of small GTPase, Rac. Mutations in DIAP1 exhibited defects in cell migration probably due to alterations in actin-dependent cellular organization [13], which was quite similar with what we observed in XIAP^−/−^ cells in the current studies. Small GTPases play important functions in a plethora of cellular events, such as regulating filamentous actin systems [39]. Rho family GTPases act as molecular switches cycling between inactive GDP-bound form in cytosol and active GTP-bound state in cytoplasm membrane [40]. RhoGDI was characterized as a down-regulator of Rho GTPases by extracting them from membranes and solubilizing them in the cytosol. RhoGDI also can interact with the switch regions of GTPases and restrict the accessibility to GEFs and GAPs so as to keep GTPase in the inactive states [39]. As we reported here, XIAP was able to physically interact with RhoGDI and inhibit its activity in regulation actin cytoskeleton assembly. So when XIAP was highly expressed, RhoGDI activity was suppressed which provided an explanation for the observations that knocking down RhoGDI in WT HCT116 cells did not affect wound closure rate since RhoGDI activity already has been inhibited by XIAP, while in XIAP^−/−^ cells where the repressive effect on RhoGDI activity was invalidate, RhoGDI knocking down exhibited much more obvious biological effects.

In addition, our studies have shown that RING domain (XIAP ΔBIR), but not BIR domains (XIAP ΔRING), could be co-immunoprecipitated in the immune complex using the antibody specific against GFP-RhoGDI. Although E3 ligase activity of RING domain was shown to be required for cell migration, impairment of its function by H467A mutation did not affect interaction with RhoGDI. So it was hypothesized that besides RhoGDI, there might be other downstream targets of E3 ligase activity of XIAP responsible for controlling cell motility, like NFκB [24] or some un-identified factors. Although E3 ligase activity of XIAP contributed to autoubiquitination of XIAP itself and ubiquitination of its binding partners, like Smac and AIF, RhoGDI was not subjected to ubiquitin conjugation even when XIAP was overexpressed.

Put together, our current studies revealed that E3 ligase activity of XIAP RING domain contributed to actin polymerization, cytoskeleton formation and cell migration. Although RING domain was required for RhoGDI interaction which mediated cell motilities, its E3 ligase activity was not involved in RhoGDI binding or ubiqutination. The alternative molecular basis for its E3 ligase activity still remains to be fully characterized.

## Materials and Methods

### Plasmids

Plasmids expressing HA-tagged XIAP, HA-XIAP ΔRING, HA-XIAP ΔBIR, HA-XIAP H467A, and pEBB-HA expression empty vector, were gifts from Dr. Colin S Duckett (University of Texas at Austin, Austin, TX) [16]. pEGFP-C3/RhoGDI vector expressing green fluorescent protein (GFP)-tagged RhoGDI and Rac1 was kindly provided by Dr. Mark R. Philips (New York University School of Medicine, New York, NY, USA). pRNA-U6/siRhoGDI and pEGFP-C3/mRhoGDI (RhoGDI gene was mutated from 403-AAA GGC GTC AAG ATT GAC-420 to 403-AAG GGA GTA AAA ATC GAT-420 to prevent destruction of exogenous mRNA by the corresponding siRNA) was provided by Dr. BL Zhang as described previously [36]. Human XIAP and RhoGDI shRNA plasmids were purchased from Open Biosystems (Pittsburgh, PA).

### Cell Culture and Transfection

Wild-type and XIAP^−/−^ HCT116 cells (human colon cancer cell lines) were kind gifts from Dr. Bert Vogelstein (Howard Hughes Medical Institute and Sidney Kimmel Comprehensive Cancer Center, The Johns Hopkins Medical Institutions, Baltimore, MD) [37]. WT and XIAP^−/−^ HCT116 cells were cultured in McCoy's 5A medium (Invitrogen, Carlsbad, CA) supplemented with 10% fetal bovine serum (FBS, Nova-Tech, Grand Island, NE) and penicillin/streptomycin (Life Technologies, Grand Island, NY). Cell transfections were performed with Lipofectamine reagent (Invitrogen) or FuGENE® HD Transfection Reagent (Roche Applied Science, Indianapolis, IN). For stable transfection, cultures were subjected to hygromycin B or G418 or puromycin (Life Technologies) drug selection, and cells surviving from the antibiotic selection were pooled as stable mass transfectants. These stable transfectants were then cultured in the selected antibiotic-free medium for at least two passages before use in experiments.

### Wound Healing Assay

Cells were seeded into each well of 6-well plates and cultured until 80% confluence. Wounds were made by sterile pipette tips. Cells were washed with serum-free PBS and then cultured in normal medium for the various time points. Photos were taken every 24 h until the wound was healed in the parental cells [41]. The wound area was quantified using the Cell Migration Analysis software (Muscale LLC, Scottsdale, AZ).

### Cell Invasion Assay

A BD BioCoatTM MatrigelTM Invasion Chamber (BD Biosciences, San Diego, CA) was used for invasion assay. Cells (2.5×10^4^) were seeded per insert in triplicate in 500 µl serum-free McCoy's 5A medium. Inserts were placed in wells containing 500 µl medium with 5% FBS and TPA (20 ng/ml). The cells were incubated for 72 h in an incubator with 5% CO_2_ humidified atmosphere. Then cells on the upper surface of the filters were first pictured and then completely removed by wiping with a cotton swab. The membrane was cut with a sharp scalpel and placed in 96-well plate. The levels of invaded/migrated cells were determined by using CellTiter-Glo® Luminescent Cell Viability Assay kit (Promega, Madison, WI) with a luminometer (Wallac 1420 Victor2 multipliable counter system) as described previously [42]. Invasion (%) = (ATP activity of invaded cells/ATP activity of migrated cells)×100%.

### Cell Proliferation Analysis

Viable cells (1×10^3^) suspended in 100 µl McCoy's 5A medium supplemented with 10% FBS were seeded into each well of 96-well plates. The plates were incubated at 37°C in a humidified atmosphere of 5% CO_2_. The cells were extracted with 50 µl lysis buffer at various time points. Cell proliferation was measured by using a CellTiter-Glo® Luminescent Cell Viability Assay kit (Promega). The results were expressed as relative proliferation rate, which was calculated as following: relative proliferation rate = ATP activity on the n^th^ day/ATP activity on 0 day.

### F-actin Content Assay

The specific cells were cultured in 10% FBS McCoy's 5A medium till 80–90% confluent. The medium was replaced with 0.1% FBS McCoy's 5A medium and incubated for 4 h. Cells were then treated with 25 ng/ml EGF for various time periods, fixed with 3.7% formaldehyde for 10 min in PBS and permeabilized with 0.1% Triton X-100 in PBS for 10 min. After washing with PBS 3 times, cells were blocked in 1% BSA/PBS at room temperature for 20 min, and then stained on a rotator with F-actin specific dye, Oregon Green 488-phalloidin (1∶40 in 1% BSA/PBS, Invitrogen), for 30 min. Cells were washed with PBS again 3 times and the bound phalloidin was extracted using 100% methanol at 4°C for 90 min. After extraction, methanol extraction was collected, and plated cells were washed with PBS 3 times and subjected to a BCA assay to determine total cell protein. Fluorescence of methanol extraction solution for each sample was recorded at 465 nm excitation and 535 nm emission by spectrophotometer, and normalized against total protein in each sample [38]. The results were expressed as relative F-actin content: F-actin(T_n_)/F-actin(T_0_) = [Fluorescence(T_n_)/mg per ml]/[Fluorescence(T_0_)/mg per ml].

Quantification of F-actin content within cells was also determined by flow cytometry according to Kobayahsi's method [43]. In brief, cells (3×10^5^) were seeded into each well of 6-well plates and cultured in 10% FBS McCoy 5A medium until 90% confluent. After stimulation with EGF for different periods of time, cells were fixed with 3.7% formaldehyde for 10 min and permeabilized with acetone for 5 min at −20°C. The cells were then blocked in 1% BSA/PBS and stained with phalloidin (3 µg/mL) for 30 min at 37°C, washed twice with PBS, re-suspended in PBS and then analyzed by flow cytometry. Relative F-actin content was expressed as an F-actin induction (averaged fluorescence of tested cells at specified time/averaged fluorescence of medium control cells).

### Immunofluorescent Staining and Confocal Microscope

HCT116 and its transfectants were cultured on cover slides in 10% FBS McCoy's 5A medium for 48 h. For EGF stimulation, the medium was replaced with 0.1% FBS McCoy's 5A medium and incubated for 4 h and then treated with EGF (25 ng/ml) for the times indicated. The cells were fixed with 3.7% paraformaldehyde for 15 min and then permeabilized with 0.1% TritonX-100 in PBS for 15 min at room temperature. The cells were then blocked with 1% BSA/PBS for 30 min, and incubated with Oregon-conjugated phalloidin for 30 min at room temperature, and then stained with 0.1 µg/ml DAPI for 1 min. The slides were washed three times with PBS and mounted with antifade reagent (Molecular Probes). The cells were observed under a confocal microscope (Leica DMI6000B). The fluorescence of cells was quantified by the software of ImageJ (version 1.37; National Institutes of Health).

### Immunoprecipitation

Cells were lysed in cell lysis buffer (1% Triton X-100, 150 mM NaCl, 10 mM Tris, pH 7.4, 1 mM EDTA, 1 mM EGTA, 0.2 mM Na_3_VO_4_, 0.5% NP-40, and complete protein cocktail inhibitors from Roche) on ice. Lysate (0.5 mg) was pre-cleared by incubation with Protein A/G plus-agarose (Santa Cruz Biotechnology, Inc.) and then incubated with specific antibody at 4°C for 2 h–12 h. Protein A/G plus-agarose (40 µl) were added to the mixture and incubated with agitation for an additional 4 h at 4°C. The immunoprecipitate was washed three times with cell lysis buffer and subjected to Western Blotting assay.

### Western Blotting

Cell extracts were prepared with cell lysis buffer (10 mM Tris-HCl, pH 7.4, 1% SDS, and 1 mM Na_3_VO_4_) and protein concentrations were determined by the protein quantification assay kit (Bio-Rad Laboratories, Hercules, CA). Thirty µg of proteins were resolved by SDS-PAGE, and subsequently probed with the indicated primary antibodies and AP-conjugated secondary antibody. Signals were detected by the enhanced chemifluorescence system as described in our previous publications [23]. The results were representative of at least three independent experiments. Antibodies against HA, XIAP (rabbit, for Western Blotting), and GFP were purchased from Cell Signaling Technology Inc (Boston, MA); against RhoGDI (Rabbit) was from Millipore (Billerica, MA); against XIAP (mouse, for IP) was from BD Science; against GADPH was obtained from Cell Signaling Technology, Inc. (Boston) or Sungene Biotech (Tianjin, China). Anti-GFP antibody-conjugated agarose beads were from Vector Laboratories, Inc. (Burlingame, CA).

### Statistical Methods

Student's t-test was utilized for determining the significance of differences. The differences will be considered significant at a p≤0.05.

## References

[1] Schimmer AD, Dalili S, Batey RA, Riedl SJ (2006). Targeting XIAP for the treatment of malignancy.. Cell Death Differ.

[2] Duckett CS, Nava VE, Gedrich RW, Clem RJ, Dongen JLV (1996). A conserved family of cellular genes related to the baculovirus iap gene and encoding apoptosis inhibitors.. EMBO J.

[3] Listen P, Roy N, Tamai K, Lefebvre C, Baird S (1996). Suppression of apoptosis in mammalian cells by NAIP and a related family of IAP genes.. Nature.

[4] Schile AJ, Garcia-Fernandez M, Steller H (2008). Regulation of apoptosis by XIAP ubiquitin-ligase activity.. Genes & Development.

[5] Jost PJ, Grabow S, Gray D, McKenzie MD, Nachbur U (2009). XIAP discriminates between type I and type II FAS-induced apoptosis.. Nature.

[6] Harlin H, Reffey SB, Duckett CS, Lindsten T, Thompson CB (2001). Characterization of XIAP-Deficient Mice.. Molecular and Cellular Biology.

[7] Burstein E, Ganesh L, Dick RD, van De Sluis B, Wilkinson JC (2004). A novel role for XIAP in copper homeostasis through regulation of MURR1.. EMBO J.

[8] Dogan T, Harms GS, Hekman M, Karreman C, Oberoi TK (2008). X-linked and cellular IAPs modulate the stability of C-RAF kinase and cell motility.. Nat Cell Biol.

[9] Li M, Song T, Yin ZF, Na YQ (2007). XIAP as a prognostic marker of early recurrence of nonmuscular invasive bladder cancer.. Chin Med J (Engl).

[10] Levkau B, Garton KJ, Ferri N, Kloke K, Nofer J-R (2001). xIAP Induces Cell-Cycle Arrest and Activates Nuclear Factor-kB: New Survival Pathways Disabled by Caspase-Mediated Cleavage During Apoptosis of Human Endothelial Cells.. Circulation Research.

[11] Kaur S, Wang F, Venkatraman M, Arsura M (2005). X-linked Inhibitor of Apoptosis (XIAP) Inhibits c-Jun N-terminal Kinase 1 (JNK1) Activation by Transforming Growth Factor b1 (TGF-b1) through Ubiquitin-mediated Proteosomal Degradation of the TGF-b1-activated Kinase 1 (TAK1).. Journal of Biological Chemistry.

[12] Deveraux QL, Reed JC (1999). IAP family proteins-suppressors of apoptosis.. Genes & Development.

[13] Srinivasula SM, Ashwell JD (2008). IAPs: What's in a Name?. Molecular Cell.

[14] Schimmer AD, Dalili S (2005). Targeting the IAP Family of Caspase Inhibitors as an Emerging Therapeutic Strategy.. Hematology.

[15] Yang YL, Li XM (2000). The IAP family: endogenous caspase inhibitors with multiple biological activities.. Cell Res.

[16] Lewis J, Burstein E, Reffey SB, Bratton SB, Roberts AB (2004). Uncoupling of the Signaling and Caspase-inhibitory Properties of X-linked Inhibitor of Apoptosis.. J Biol Chem.

[17] Kleinberg L, Flørenes VA, Nesland JM, Davidson B (2007). Survivin, a member of the inhibitors of apoptosis family, is down-regulated in breast carcinoma effusions.. Am J Clin Pathol.

[18] Kleinberg L, Flørenes VA, Silins I, Haug K, Trope CG (2007). Nuclear expression of survivin is associated with improved survival in metastatic ovarian carcinoma.. Cancer.

[19] Kluger H, McCarthy M, Alvero A, Sznol M, Ariyan S (2007). The X-linked inhibitor of apoptosis protein (XIAP) is up-regulated in metastatic melanoma, and XIAP cleavage by Phenoxodiol is associated with Carboplatin sensitization.. Journal of Translational Medicine.

[20] Akyurek N, Ren Y, Rassidakis GZ, Schlette EJ, Medeiros LJ (2006). Expression of inhibitor of apoptosis proteins in B-cell non-Hodgkin and Hodgkin lymphomas.. Cancer.

[21] Nemoto T, Kitagawa M, Hasegawa M, Ikeda S, Akashi T (2004). Expression of IAP family proteins in esophageal cancer.. Experimental and Molecular Pathology.

[22] Nagi C, Xiao G-Q, Li G, Genden E, Burstein DE (2007). Immunohistochemical detection of X-linked inhibitor of apoptosis in head and neck squamous cell carcinoma.. Annals of Diagnostic Pathology.

[23] Liu J, Zhang D, Luo W, Yu Y, Yu J (2011). X-linked Inhibitor of Apoptosis Protein (XIAP) Mediates Cancer Cell Motility via Rho GDP Dissociation Inhibitor (RhoGDI)-dependent Regulation of the Cytoskeleton.. Journal of Biological Chemistry.

[24] Mehrotra S, Languino L, Raskett C, Mercurio A, Dohi T (2010). IAP regulation of metastasis.. Cancer Cell.

[25] Fidler IJ (1990). Critical Factors in the Biology of Human Cancer Metastasis: Twenty-eighth G. H. A. Clowes Memorial Award Lecture.. Cancer Research.

[26] Lin M, van Golen KL (2004). Rho-Regulatory Proteins in Breast Cancer Cell Motility and Invasion.. Breast Cancer Research and Treatment.

[27] del Peso L, Hernández-Alcoceba R, Embade N, Carnero A, Esteve P (1997). Rho proteins induce metastatic properties in vivo.. Oncogene.

[28] Wittmann T, Waterman-Storer CM (2001). Cell motility: can Rho GTPases and microtubules point the way?. Journal of Cell Science.

[29] Kjoller L, Hall A (1999). Signaling to Rho GTPases.. Experimental Cell Research.

[30] Adra CN, Manor D, Ko JL, Zhu S, Horiuchi T (1997). RhoGDIg: A GDP-dissociation inhibitor for Rho proteins with preferential expression in brain and pancreas.. Proceedings of the National Academy of Sciences.

[31] Miura Y, Kikuchi A, Musha T, Kuroda S, Yaku H (1993). Regulation of morphology by rho p21 and its inhibitory GDP/GTP exchange protein (rho GDI) in Swiss 3T3 cells.. Journal of Biological Chemistry.

[32] Leffers H, Nielsen MS, Andersen AH, Honore B, Madsen P (1993). Identification of Two Human Rho GDP Dissociation Inhibitor Proteins Whose Overexpression Leads to Disruption of the Actin Cytoskeleton.. Experimental Cell Research.

[33] Fong WG, Liston P, Rajcan-Separovic E, St Jean M, Craig C (2000). Expression and genetic analysis of XIAP-associated factor 1 (XAF1) in cancer cell lines.. Genomics.

[34] Yang L, Cao Z, Yan H, Wood WC (2003). Coexistence of High Levels of Apoptotic Signaling and Inhibitor of Apoptosis Proteins in Human Tumor Cells: Implication for Cancer Specific Therapy.. Cancer Res.

[35] Staiger CJ, Blanchoin L (2006). Actin dynamics: old friends with new stories.. Current Opinion in Plant Biology.

[36] Zhang B, Zhang Y, Dagher M-C, Shacter E (2005). Rho GDP Dissociation Inhibitor Protects Cancer Cells against Drug-Induced Apoptosis.. Cancer Res.

[37] Cummins JM, Kohli M, Rago C, Kinzler KW, Vogelstein B (2004). X-Linked Inhibitor of Apoptosis Protein (XIAP) Is a Nonredundant Modulator of Tumor Necrosis Factor-Related Apoptosis-Inducing Ligand (TRAIL)- Mediated Apoptosis in Human Cancer Cells.. Cancer Res.

[38] Taniuchi S, Kinoshita YO, Yamamoto A, Fujiwara T, Hattori K (1999). Heterogeneity in F-actin polymerization of cord blood polymorphonuclear leukocytes stimulated by N-formyl- methionyl-leucyl-phenylalanine.. Pediatrics International.

[39] Dovas A, Couchman JR (2005). RhoGDI: multiple functions in the regulation of Rho family GTPase activities.. Biochem J.

[40] DerMardirossian C, Bokoch GM (2005). GDIs: central regulatory molecules in Rho GTPase activation.. Trends in Cell Biology.

[41] Shan D, Chen L, Njardarson JT, Gaul C, Ma X (2005). Synthetic analogues of migrastatin that inhibit mammary tumor metastasis in mice.. Proceedings of the National Academy of Sciences of the United States of America.

[42] Ouyang W, Zhang D, Li J, Verma UN, Costa M (2009). Soluble and insoluble nickel compounds exert a differential inhibitory effect on cell growth through IKKα-dependent cyclin D1 down-regulation.. Journal of Cellular Physiology.

[43] Chan AY, Raft S, Bailly M, Wyckoff JB, Segall JE (1998). EGF stimulates an increase in actin nucleation and filament number at the leading edge of the lamellipod in mammary adenocarcinoma cells.. J Cell Sci.

